# Measuring the incidence and reporting of violence against women and girls in liberia using the 'neighborhood method'

**DOI:** 10.1186/1752-1505-7-20

**Published:** 2013-09-23

**Authors:** Lindsay Stark, Ann Warner, Heidi Lehmann, Neil Boothby, Alastair Ager

**Affiliations:** 1 Program on Forced Migration and Health, Mailman School of Public Health, Columbia University, 60 Haven Avenue, New York, NY 10032, USA; 2 International Rescue Committee, New York, NY 10018, USA

**Keywords:** Liberia, Gender-based violence, Rape, Child protection

## Abstract

**Background:**

This paper reports on the use of a “neighborhood method” to measure the nature and incidence of violence against women and girls in post-conflict Liberia.

**Methods:**

The study population comprised females in Montserrado and Nimba counties. Study participants were randomly selected for interviews using multi-stage cluster sampling. 30 clusters of households were sampled in each county. Information on incidents of domestic violence and rape within the preceding 18 months was collected with regard to females of all ages in the respondent’s household, and those of her four closest neighbors to make up the full sample.

**Findings:**

Households in the sample contained 7015 females (1687 girls, 4586 women, 742 age missing) in Montserrado and 6632 (2070 girls, 4167 women, 95 age missing) in Nimba. In the previous 18 months 54.1% (CI 53.1-55.1) and 55.8% (CI 54.8-56.8) of females in Montserrado and Nimba respectively were indicated to have experienced non-sexual domestic abuse; 19.4% (CI 18.6-20.2) and 26.0% (CI 25.1-26.9) of females in Montserrado and Nimba respectively to have been raped outside of marriage; and 72.3% (CI 70.7-73.9) and 73.8% (CI 72.0-75.7) of married or separated women in Montserrado and Nimba respectively to have experienced marital rape. Husbands and boyfriends were reported as the perpetrators of the vast majority of reported violence. Strangers were reported to account for less than 2% of the perpetrators of rape in either county. Incidents were most commonly disclosed to other family members or to friends and neighbors, and less often to formal authorities such as the police, court or community leaders. Incidents were approaching fifty times more likely to be reported to police if perpetrated by strangers rather than intimate partners.

**Conclusions:**

Violence against women and girls is widespread in the areas studied. Programming needs to address the fact that this violence is primarily occurring in the household, where most incidents go unreported outside the immediate family or social circle. Police and hospital reports severely under-represent these known perpetrators. Inter-interviewer variance and differences in reports for self and neighbors for some outcomes caution the precision and validity of some estimates. However, the potential utility of the neighborhood method for estimating prevalence rates with an accuracy suitable for programmatic purposes in conflict-affected and post-conflict settings is noted.

## Background

Prior to the 1990s, gender-based violence during armed conflict was largely viewed as a regrettable but inevitable byproduct of war [1]. However, over the last 20 years humanitarian organizations have increasingly recognized that violence against women and girls is a critical public health issue, a violation of human rights, and an obstacle to reconstruction and development of conflict-affected countries [2].

While services for survivors have increased in many places, the science of measuring and understanding violence against women and girls is less developed, especially in conflict-affected settings [2,3]. Most data collected by non-governmental organizations relies on non-representative samples, based on service delivery statistics gathered when women or girls seek health, psychosocial or other services after the incident [4]. Because many survivors do not seek such services, the prevalence of violence is potentially grossly underestimated [5]. The lack of data of the magnitude of violence against women and girls hampers prevention and response efforts, and contributes to the impunity that may fuel further violence.

Documenting physical violence and rape is difficult anywhere, and is particularly challenging in settings where infrastructure and social fabric have been eroded by conflict [2,3,6]. The enormous sensitivity of the issues, fear of recrimination by survivors, cultural permissiveness, stigma, and the lack of reporting mechanisms can all impede the collection of population-based data [3].

All of these factors are at work in the recent context of Liberia. A series of civil wars from 1989 to 2003 destroyed Liberia’s social fabric and infrastructure. 270,000 people died, either from violence or from illnesses that went untreated because war disrupted their access to life-saving medical facilities and medicines. More than 700,000 people fled Liberia, and more than 1.4 million people were displaced within the country [7]. Women and girls bore the brunt of the hardship. In addition to being deprived of education and basic healthcare, large numbers of women and girls suffered from sexual violence. A small-scale study conducted in Monrovia found that 49 percent of female participants had experienced at least one act of physical or sexual violence by a soldier or fighter [6]. A study conducted in 2003 found that 75 percent of the Liberian female refugees in three refugee camps in Sierra Leone interviewed had been victims of sexual abuse prior to their displacement (Benton, A: *Research Report: Prevalence of Gender-based Violence Among Liberian Women in Three Refugee Camps,* unpublished data). 55 percent of them reported suffering further abuse since their displacement.

What is the legacy of such rates of gender-based violence in post-conflict Liberia? When Ellen Johnson-Sirleaf was inaugurated as President of Liberia in January 2006, one of her first executive acts was to enact a new rape law. While this new legislation strengthened the legal standing for survivors of rape, there appear many obstacles to its full implementation [8]. There has been a lack of population-based data on the occurrence of rape since the end of the war, despite concerns that – while risks of politically or militarily motivated attacks may be much reduced – the social and economic impact of prolonged conflict may exacerbate risks of other forms of sexual violence [2].

The current study attempted to provide a valid and reliable estimate of the incidence of violence against women and girls in Liberia. The research was implemented by the Program on Forced Migration & Health at Columbia University in partnership with the International Rescue Committee’s (IRC) Gender Based Violence program in the summer of 2007. The geographical focus of the study was in two counties within which IRC were delivering services for survivors of domestic violence and sexual assault. Research objectives included: contributing assessment information regarding the incidence and nature of violence against women and girls in IRC’s operational areas in two counties; investigating reporting and disclosure patterns; and establishing a baseline against which to measure change over time.

## Methods

### Participants

The study population was females of all ages in Montserrado and Nimba counties. Monsterrado is an urban county on the Atlantic coast that is home to Liberia’s capital, Monrovia. Nimba County is a more rural county to the northeast of Monsterrado, bordered by Guinea and Cote d’Ivoire. Study participants were randomly selected using multi-stage cluster sampling. Adult women identified as female heads of household gave verbal consent to trained interviewers before the interview process began. They were interviewed about their own experiences with violence, and also that of the other females in their households and the females in the households of their four closest neighbors. To preserve anonymity no identifying information was collected on the respondent or any of the other households she discussed.

### Study design

The study used the “Neighborhood Method” [9,10] which, drawing on some of the principles of the “Sisterhood Method” [11], randomly selects women to interview and asks them not only about their experiences within their own household, but also of selected neighbors. This method increases the power of studies limited by resources, logistics and security by collecting information on many women from each interview. Such potential for power and efficiency is particularly pertinent in humani-tarian contexts where prompt data collection may be an ethical and pragmatic imperative. Here interviews gathered information on: 1) the respondent herself; 2) other women and girls (females under the age of 18) in the respondent’s household; 3) female heads of household in the four closest neighboring households; and 4) other women and girls in those neighboring households. The time frame covered was January 2006 through the day of the interview (approximately 18 months). To aid recall, the interviewers used the inauguration of the new President (Ellen Johnson-Sirleaf) as the start date of the recall period.

The research protocol, interviewer guide and data collection forms were adapted in Liberia from a similar study undertaken in northern Uganda [9]. The questionnaire was field-tested in Monrovia, and the sampling and interviewing techniques were further piloted in the field. Data collection lasted for four weeks. The research was conducted under Columbia University Medical Center’s Institutional Review Board (IRB) approval AAAB7134.

### Primary outcomes

The interview covered three main areas of violence, which – drawing upon relevant national legal definitions - were defined as follows:

▪ *Domestic violence*: Any act of (non-sexual) physical violence perpetrated by an intimate partner or family/household member.

▪ *Rape*: the intentional penetration or attempted penetration of another person’s vagina, anus, mouth or any other opening without the victim’s consent.

▪ *Marital rape*: the intentional penetration or attempted penetration of another person’s vagina, anus, mouth or any other opening without the victim’s consent by a partner in the context of marriage.

### Sampling

Multi-stage cluster sampling was used to select households. Because a census had last been completed in the 1980s, population figures were drawn from IRC records along with updated information from the Liberia Institute of Statistics and Geo-Information Services. These figures were further verified and modified during the early phases of research based on population information from local officials and household counting.

For sampling purposes, sought event prevalence was assumed to be 5%, setting a precision of 2% and a design effect of 2. This sought prevalence was a conservative estimate drawn from a structured review of prevalence studies of gender-based violence in humanitarian settings [3]. Based on each respondent providing data on at least six other adult females, a minimum sample size of 300 adult female heads of household was determined using EpiInfo 6.0 [12]. With the anticipation of refusal of consent and other sampling losses, a sample of 330 participants in 30 clusters was targeted in each county.

Clusters were selected proportional to population size of the communities that made up each county. If a community was randomly assigned one or more clusters, that community was then sub-divided into segments of 200 or fewer households using a combination of simple mapping and global positioning satellite (GPS) technology. Clusters were then randomly selected from these segments. A random number sequence was used to select the starting household within each selected segment. Interviewers followed a fixed sampling interval within each segment, mapping the houses in advance to avoid duplication. Generally, two or three interviewers worked in each cluster, assigned on the basis of a combination of random assignment and availability. Each interviewer conducted between three and five interviews per day.

### Interview

Interviews were conducted by six female IRC social workers who had a minimum of one year’s experience working with survivors of gender-based violence. There were two interviewers from each of IRC’s operational counties. A single interviewer approached the selected house and asked to speak to the female head of the household^a^. She explained the purpose of the interview, its anticipated duration, the assurance of anonymity and the need for privacy. If the woman identified as female head of household gave her informed verbal consent, the interview began in a private location chosen by the respondent. If the woman refused or was unable to speak to the interviewer privately, the interviewer thanked her for her time and moved to the next house.

The interviewer first asked for basic demographic information (age and marital status) about the respondent, other women and girls in her household, her four closest female neighbors (identified by location by the interviewer) and all the women and girls in those four households. She then asked the woman to describe some of the biggest problems facing women and girls in her community. The interviewer then asked about incidents of physical violence in the interviewee’s neighborhood, if the topic had not already been raised by the interviewee. The interviewer then asked about the first neighbor and the other females living in that household, and whether any of them had experienced domestic violence or sexual abuse since January 2006. The interviewers used local terminology to discuss these sensitive topics and elicited “yes,” “no,” or “don’t know” responses. If an incident was reported to have occurred, the interviewer asked for information about the perpetrator (relationship to survivor, approximate age), whether or not the survivor had reported the incident, and if so, to whom (if reports were made to several audiences, the principal, most formal audience used was recorded). She also recorded additional information in narrative form. She used the same format to ask about the other women and girls in those households. She finally asked about the woman herself and other women and girls in her household.

Interviews averaged 40 minutes in length. Privacy and confidentiality were paramount concerns. Interviewers took care to assure participants that no names were being recorded, and that there would not be compensation for their participation, nor consequences if they refused to answer any questions. At the end of the interview, the interviewers provided information on relevant health, legal and social services in their area.

### Data collection and analysis

Data was recorded by the interviewer onto individual data sheets during the interview (using 'post-it’ notes to record first names, which were then removed at the end of the interview to preserve confidentiality). Data sheets were passed to the research coordinator at the end of the day for review. The research coordinator entered the data into Microsoft Excel. Point estimates of incidence of key variables were calculated using Epi Info 6.0 [12], with confidence intervals adjusted for clustering. The Statistical Package for Social Sciences (SPSS) version 14.0 [13] was used to conduct analyses, which took account of the cluster design of the study. Independent t-tests and analysis of variance (ANOVA) were used to analyze mean differences in continuous variables. Pearson’s chi-square tests were used to measure proportions in categorical variables. Logistic regression was used to conduct multivariate analyses on dichotomous outcomes. Inter-cluster correlations were examined across multiple variables to estimate an *a posteriori* design effect for calculation of appropriate confidence intervals.

## Results

### Participation rates

In Montserrado County, of 332 households approached, 18 (6%) were unavailable and 14 (4%) refused to participate. In Nimba County, 28 of 342 households approached were unavailable (9%) and 14 refused to participate (5%).

### Demographic characteristics of sample

Table 1 summarizes key demographic characteristics of the resultant sample. From 300 respondents in each county, data was collected with respect to 7,015 females in Montserrado County and 6,632 females in Nimba County. An average of 4.7 females and 4.3 females were reported per household in Montserrado County and Nimba County respectively.

**Table 1:** Characteristics of sample population (all Female)

	**Montserrado county**	**Nimba county**
**Sample Size**	7,015	6,632
▪ Respondents	300	300
▪ Other females in respondents’ household	1,282	1,178
▪ Neighbors & other females in neighbors’ household	5,433	4,854
**Average age**	26.3	24.4
▪ Percentage under 18	24.0%	32.7%
▪ Percentage 18 and older	65.4%	65.8%
▪ Age Missing	10.6%	1.5%
**Marital Status**		
▪ Single	61.2%	69.8%
▪ Married	31.8%	24.1%
▪ Separated/Divorced	1.3%	1.2%
▪ Widowed	5.6%	4.8%

### Awareness of violence

Of primary respondents in Montserrado County, 279 (95.2%) noted domestic violence without prompting when asked to name some of the biggest problems facing women in their communities. 124 (44.1%) cited rape without prompting. In Nimba County, 267 (92.7%) of primary respondents noted domestic violence, and 94 (33.2%) rape, without prompting (Table 2).

**Table 2:** Rates of those experiencing specified forms of violence, January 2006-July 2007

	**Montserrado county**			**Nimba county**		
**Incident**	N*	**Proportion**	**CI**	N*	**Proportion**	**CI**
**Any Violence**	7015	56.1%	(55.1-57.1)	6632	58.7%	(57.7-59.7)
▪ Under 18	1687	24.2%	(22.5-25.9)	2070	19.4%	(18.0-20.8)
▪ 18 and older	4585	76.7%	(75.7-77.7)	4167	78.4%	(77.4-79.4)
**Domestic Violence**	6849	54.1%	(53.1-55.1)	6281	55.8%	(54.8-56.8)
▪ Under 18	1675	15.0%	(13.6-16.4)	2060	14.4%	(13.2-15.7)
▪ 18 and older	4554	75.5%	(74.5-76.5)	4155	76.8%	(75.7-77.9)
**Rape outside Marriage**	6665	19.4%	(18.6-20.2)	6096	26.0%	(25.1-26.9)
▪ Under 18	1658	13.5%	(12.2-14.9)	2045	11.0%	(9.9-12.1)
▪ 18 and older	4387	24.3%	(23.3-25.3)	3992	33.8%	(32.6-35.0)
Marital Rape**	2029	72.3%	(70.7-73.9)	1467	73.8%	(72.0-75.7)

* valid N for reporting (taking into consideration missing data).

** of married, separated & divorced women.

### Incidence of violence

56.1% (55.1-57.1) of the entire sample population in Montserrado County and 58.7% (57.7-59.7) of the sample population in Nimba County had experienced at least one act of violence in the previous 18 months. 75.5% (74.5-76.5) of adult females in Montserrado and 76.8% (75.7-77.9) in Nimba had experienced at least one act of domestic violence in the previous 18 months. Among adult females, 24.3% (23.3-25.3) in Montserrado and 33.8% (32.6-35.0) in Nimba had experienced rape or attempted rape outside of marriage. Among married, separated and divorced women, 72.3% (70.7-73.9) in Montserrado and 73.8% (72.0-75.7) in Nimba were reported as having experienced marital rape in the reporting period.

Domestic violence had been experienced by 15.0% (13.6-16.4) of girls in Montserrado County and 14.4% (13.2-15.7) of girls in Nimba County. 13.5% (12.2-14.9) of girls in Montserrado and 11.0% (9.9-12.1) in Nimba had experienced an act of rape or attempted rape in the previous 18 months.

### Identity of perpetrator

In both counties, husbands or boyfriends – together referred to as “intimate partners” – were the reported perpetrators of more than 95% of reported domestic violence. Combining categories of rape outside of marriage and marital rape, violence by husbands and boyfriends accounted for the vast majority of reported assaults (91.6% in Montserrado and 94.1% in Nimba). Neighbors or community members were reported as perpetrators of 3.5% and 2.8% of rapes in Montserrado and Nimba respectively. Strangers accounted for less than 2% of the perpetrators of rape in both counties. The most frequent perpetrators of rape against girls were neighbors and community members (35.0% Montserrado, 24.7% Nimba) or boyfriends (32.3% in Montserrado; 50.6% in Nimba). Strangers were reported as the perpetrators of 10.2% and 4.8% of rapes against girls, but only 1.2% and 0.7% of perpetrators of rape against adult women, in Montserrado and Nimba respectively.

### Reporting patterns

Domestic violence was the type of violence that was most commonly reported to someone else (93.1% and 96.4% of incidents, in Montserrado and Nimba respectively); marital rape was least commonly reported (58.8% and 63.0% of incidents, in Montserrado and Nimba respectively). The latter figure indicates that neighbors’ knowledge of marital rape was frequently by witnessing or hearing incidents, rather than directly being told. Table 3 presents the probability of incident reports that are made being principally directed to any given audience. Domestic violence incidents were most commonly principally reported to family members, while (non-marital) rape was most commonly reported to friends. Rape was reported to more formal audiences (police, courts, health posts, community leaders, etc.) less than 10% of the time.

**Table 3:** Probability of principal reports made being to particular audience by form of violence

	**Montserrado**			**Nimba**		
**Reporting audience**	**Domestic violence**	**Marital rape**	**Non-marital rape**	**Domestic violence**	**Marital rape**	**Non-marital rape**
	**N = 3410**	**N = 855**	**N = 1136**	**N = 3339**	**N = 677**	**N = 1235**
Family Member	0.699	0.368	0.255	0.686	0.270	0.209
Friend or Neighbor	0.267	0.615	0.634	0.274	0.706	0.710
Community Leader	0.003	0.001	0.008	0.008	0.009	0.012
Police/Court	0.023	0.006	0.097	0.018	0.001	0.062
Religious Leader	0.005	0.011	0.001	0.012	0.013	0.002
Hospital/Clinic	0.000	0.000	0.004	0.001	0.000	0.002
Other	0.004	0.005	0.003	0.005	0.005	0.004

Minors were significantly more likely than adult women to report non-marital rape. (34.7% vs. 4.4%, p<.0001, and 24.6% vs. 2.3%, p<.001, Montserrado and Nimba respectively). In Montserrado County, if the perpetrator was an intimate partner, the case was reported to the police 1.0% of the time, in comparison to 47.8% if the perpetrator was not an intimate partner (p<.0001). In Nimba County, the equivalent percentages were 0.6% and 49.6% (p<.0001).

### Variance between self and secondary reporting

To investigate potential biases between self and secondary reporting, we compared reports of respondents on their own experience with their reports on the female head of neighboring households. There were no differences in reported rates of marital rape and non-marital rape between respondents themselves and female heads of neighboring households (see Table 4). There was a non-significant trend for higher rates of domestic violence to be reported by respondents. However, the respondent selection method appears in both counties to have identified respondents significantly younger (mean age 35.2 vs. 38.3, p<0.01 in Montserrado, 31.7 vs. 36.5, p<0.001 in Nimba) than the identified female heads of households in neighboring households. When controlled for age and marital status, differences between respondents and neighbors in rates of reported domestic violence were, in both counties, eliminated.

**Table 4:** Indicated rates for respondent and senior females of neighboring households

	**Montserrado**			**Nimba**		
	**Respondent**	**Neighbor**	**P**	**Respondent**	**Neighbor**	**P**
Domestic Violence	76.7% (n=296)	69.2% (n=1170)	.116	83.3% (n=300)	75.7% (n=1178)	.082
Marital Rape	73.5% (n=163)	74.7% (n=640)	.846	69.0% (n=152)	74.9% (n=536)	.366
Non-Marital Rape	20.0% (n=295)	20.6% (n=1130)	.888	23.4% (n=299)	26.8% (n=1135)	.460

**Figure 1:**
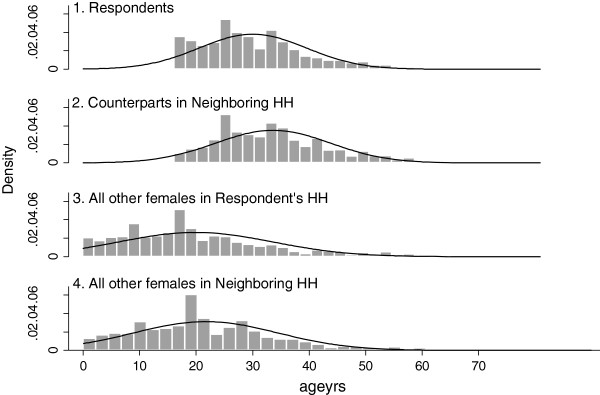
Distribution of reported age.

The age distribution of other females in the respondent’s household and non-female head of household females in the neighboring household were equivalent (p=.299). Figure 1 presents a histogram of the age distribution of respondents, their counterparts in neighboring households, and all other females reported on in the respondent’s household and neighboring households. The latter distributions are notably skewed compared with what one would expect from a population pyramid in Liberia, with a smaller than expected number of young girls being reported upon.

### Interviewer variance

There was significant inter-interviewer variance in reported rates for each outcome. Overall, rates ranged from 31% to 78% for domestic violence, from 28% to 83% for marital rape, and from 17% to 44% for non-marital rape respectively. While real variations in womens’ experience of violence across households may be reflected here, an ICC (intra-cluster correlation) of 0.18 with respect to interviewer (the largest such correlation determined, all others were below 0.08) indicates the potential for substantive differences in the reliability of interviewers in eliciting reports of violence. The *a posteriori* design effect calculated on the basis of this ICC was 2.61, and all confidence intervals and p values cited were calculated using this figure.

## Discussion

### Prevalence of violence

Four years after the end of the war, violence against women and girls was still widespread in Liberia. More than half of the female population was indicated to have experienced some form of violence in the preceding 18 months. More than 75 percent of adult women were indicated to have experienced domestic violence, and between one quarter and one third of adult women were indicated as having been raped outside of marriage, in the same period. Among married (and separated and divorced) women over 70% were indicated to have experienced marital rape during this interval. Marriage is identified as a risk factor for both sexual and non-sexual violence. Husbands and boyfriends were reported as the perpetrators of the vast majority of reported violence, but incidents were approximately fifty times more likely to be reported to police if perpetrated by strangers rather than intimate partners.

Violence against girls is also prevalent among the sample population. One in 4 girls was reported as having experienced some form of violence, with more than 1 in 10 girls reported as being raped. It was more common for rape of a girl to be reported to the police or court, but this still occurs less than half of the time. While the case definition for domestic violence adopted included any act of physical abuse in the home, the focus of questions was not explicitly on the treatment of children and cultural norms of discipline, and are likely to have resulted in an under-reporting of child abuse in the household involving non-sexual physical violence. Furthermore, all reports of abuse of children relied on secondary reports, which is also likely to have resulted in an under-estimate of actual incidence.

### Methodological issues

Assessing the suitability of the neighborhood method as a methodological approach to the estimation of health issues and human rights violations requires consideration of the realities of the humanitarian context. There is a widespread perception in the field of humanitarian aid that valuable time and money for life-saving assistance would be lost if rigorous assessment and evaluation methodologies were put into place. As science struggles to find its place in a context where funds are limited and programs need to be quickly established, developing assessment and evaluation methods that can be implemented cheaply and quickly are vital.

The neighborhood method was designed to be able to estimate – within confidence intervals suitable for guiding programmatic response - population-based prevalence at a fraction of the cost and time that would be required for a full household survey. We acknowledge that, even with the development of new technologies supporting rapid data capture, it is extremely unlikely that implementation of such an expensive and time-intensive exercise as a full household survey will become common practice in agencies’ program design and evaluation for such issues as gender-based violence. Approaches such as the neighborhood method may, in consequence, have an important role in such contexts.

Neighbors were initially hypothesized to be good key informants on the grounds of the close proximity of living quarters in camps for refugees and for internally displaced persons (IDPs) that generally are present in conflict-affected settings. This overcrowding has been known to create a lack of privacy that results in neighbors knowing intimate details about one another. This study was the first attempt to use the neighborhood method outside of a refugee camp setting, and it was uncertain if women would be comfortable and confident in reporting in less constrained physical settings. Field experience, and our findings, largely suggest that women were knowledgeable about - and willing to share information regarding - their neighbors, but there were some limitations to the method in this setting. Although some differences between self- and secondary report are eliminated when controlling for age and marital status, disclosure patterns suggest that respondents were moderately more able or more willing to disclose information about their own experiences with violence than that of their neighbors, especially with regard to domestic violence. The interviews were conducted in or just outside the respondent’s home, which was deemed the most practical venue for interviews by the research team, but may have contributed to respondents’ reluctance to share information on neighbors when they could feasibly see or hear the conversation. In a country with 85% unemployment [7], people spend a great deal of time in the home, and at times it was difficult to identify a private place within the home where an interview could be conducted. Overall, we consider the difference between self-reporting and secondary reporting to be within an “acceptable” threshold (less than 10% for all outcomes). The net effect of disclosure biases remains a likely underestimate of the actual level of violence in these communities.

The interview method aimed to blend both qualitative and quantitative methodologies in its semi-structured format. This method allowed for interviewers to build trust and rapport with the respondent, so that they would feel both safe and comfortable disclosing highly sensitive information about themselves and their neighbors. Most of the time, respondents themselves raised the issues of violence and sexual violence allowing for the topics to more easily enter the conversation.

Ethical considerations need to be paramount in research on violence against women and girls. The biggest risk of this research is that women would be put at increased risk for talking about the violence they faced. The research protocol sought to mitigate this risk by introducing the survey as one about “issues facing women in the community” (rather than a gender-based violence survey), insisting on privacy as much as possible, and gathering no identifying information on respondents. Furthermore, research was only conducted in areas where IRC was providing services, and interviews were conducted by trained social workers. Interviewers gave information and referrals to relevant health, legal and social services at the end of the interview. In some cases, where the survivor requested and agreed, follow-up was provided by a social worker immediately.

### Limitations

There are a number of methodological limitations that are appropriate to highlight. First, attempting to undertake a population-based survey is difficult in an area where conflict has caused extensive displacement and impeded the ability of government institutions to collect even basic demographic information. The last census conducted prior to this study was in 1983, making it extremely challenging to obtain reliable population information.

In addition, identifying clear-cut, culturally sensitive case definitions, even for concepts such as 'marriage’ was challenging. The research would have benefitted from more time spent at the outset exploring key concepts in depth. Focus group discussions, for example, with the target population on their understanding of physical and sexual violence might have helped interviewers to probe and code these issues with greater consistency.

Household sizes suggested by the study are somewhat larger than anticipated from other data sources. Although total household size was not recorded on visits to households, proration from recorded female household membership on the basis of estimated sex ratios suggests an average household size of between 8 and 9 persons compared to 4.7 and 5.9 persons for Montserrado and Nimba respectively as indicated by the 2008 national census [14]. While the sampling strategy adopted may have resulted in greater probability of respondents being selected from more populous households (on the basis of their being more likely at home during survey periods), this would not account for more populous neighbor households being selected. It is likely, therefore, that respondents adopted a more inclusive definition of household membership than the *de jure* standard applied in the national census. Average reported age was also higher than the national average documented in the 2008 census (25 years versus 22 years) [15]. Although this may in part result from inter-county variation, Figure 1 suggests this is most plausibly attributable to trends for respondents to be more inclusive in reporting on adolescent and adult women compared to younger girls and to provide somewhat inflated estimates of the ages of female heads of household in neighboring homes. Such challenges are familiar in the context of Liberia with the age pyramid suggested from the 2007 national DHS [16] judged to be 'implausible' (p. 8) due to systematic misreporting.

While one of the perceived strengths of the neighborhood method is that it uses a semi-structured interview format, which is intended to build trust and rapport and encourage disclosure, it is also clear that this type of interview format requires a more advanced skill-set from interviewers and is also likely more prone to interviewer variation than a standard household survey. The variation between interviewers in terms of rates reported for this study suggests some were more skilled than others in reliably eliciting reports of experienced violence. In future use of this methodology it will be important to place major emphasis upon – and monitor procedures with respect to - consistency of interviewing on delicate issues requiring sensitive (and thus skilled) probing.

## Conclusions

Our findings demonstrate that violence against women and girls was widespread in studied communities in Liberia. Past research in Liberia has focused on conflict-related sexual violence by armed combatants, but this study shows that some four years after the end of the civil war, women and girls were most at risk of experiencing violence inside their own homes. Hospital and police records mask this fact and appear to disproportionately record rape by strangers and rape of young victims. Organizations addressing gender-based violence need to design their prevention, response and advocacy efforts to reflect the true population-based reality. When the Rape Law was passed in Liberia in 2006, marital rape was left out of the legislation [L]. The findings of this research strongly encourage reconsideration of this decision.

The neighborhood method appears to be a promising tool for measuring violence in some conflict-affected settings [9,10]. By establishing rapport and confidentiality with one respondent, using established principles of qualitative interviewing [14], information is gained on four other households (an average of 20 females in the current study), achieving the statistical power usually limited to quantitative surveys. This is especially valuable in settings where resources and data are scarce, which is typical in conflict-affected and post-conflict environments. In such settings, ethical considerations remain paramount, however, and future work needs to consider the parameters that render data collection regarding neighbors reliable, valid and culturally acceptable.

### Endnote

^a^Interviews were one-on-one, except in one community in Monrovia, where interviewers worked in pairs for security reasons.

## Competing interests

The authors declare that they have no competing interests.

## Authors’ contributions

LS and AA participated in the design of the study. HL facilitated fieldwork engagement and provided technical guidance on implementation. AW coordinated data collection. AA supervised preparation of the initial draft report by AW and coordinated authors’ inputs during the course of revisions. LS supported data analysis. NB served as Principal Investigator and AA as Research Director, respectively, of the CPC initiative of which this work was part. All authors engaged in development of the final draft of the manuscript. All authors read and approved the final manuscript.
